# Interventions for reducing self-stigma in people with mental illnesses: a systematic review of randomized controlled trials

**DOI:** 10.3205/000248

**Published:** 2017-04-24

**Authors:** Roland Brian Büchter, Melanie Messer

**Affiliations:** 1 Institute for Quality and Efficacy in Health Care (IQWiG), Cologne, Germany; 2 Faculty for Health Sciences, Health Services Research and Nursing Science, University of Bielefeld, Germany

**Keywords:** stigma, mental illness, systematic review, anti-stigma interventions, anti-stigma booklet

## Abstract

**Background:** Self-stigma occurs when people with mental illnesses internalize negative stereotypes and prejudices about their condition. It can reduce help-seeking behaviour and treatment adherence. The effectiveness of interventions aimed at reducing self-stigma in people with mental illness is systematically reviewed. Results are discussed in the context of a logic model of the broader social context of mental illness stigma.

**Methods:** Medline, Embase, PsycINFO, ERIC, and CENTRAL were searched for randomized controlled trials in November 2013. Studies were assessed with the Cochrane risk of bias tool.

**Results:** Five trials were eligible for inclusion, four of which provided data for statistical analyses. Four studies had a high risk of bias. The quality of evidence was very low for each set of interventions and outcomes. The interventions studied included various group based anti-stigma interventions and an anti-stigma booklet. The intensity and fidelity of most interventions was high. Two studies were considered to be sufficiently homogeneous to be pooled for the outcome self-stigma. The meta-analysis did not find a statistically significant effect (SMD [95% CI] at 3 months: –0.26 [–0.64, 0.12], I^2^=0%, n=108). None of the individual studies found sustainable effects on other outcomes, including recovery, help-seeking behaviour and self-stigma.

**Conclusions:** The effectiveness of interventions against self-stigma is uncertain. Previous studies lacked statistical power, used questionable outcome measures and had a high risk of bias. Future studies should be based on robust methods and consider practical implications regarding intervention development (relevance, implementability, and placement in routine services).

## Zusammenfassung

**Hintergrund:** Von Selbststigmatisierung spricht man, wenn Menschen mit psychischen Erkrankungen negative Stereotypen und Vorurteile gegenüber ihrer eigenen Erkrankung verinnerlichen. Mögliche Folgen sind eine reduzierte Inanspruchnahme von Behandlungs- und Unterstützungsleistungen und eine niedrigere Behandlungsadhärenz. In dieser Arbeit wird die Wirksamkeit von Interventionen analysiert, die eine Senkung der Selbststigmatisierung zum Ziel haben. Die Ergebnisse werden anhand eines Logic Models in einen breiteren sozialen und gesellschaftlichen Kontext eingebettet und im Rahmen dessen diskutiert.

**Methoden:** Es wurde eine Literaturrecherche nach randomisierten Studien in Medline, Embase, PsycINFO, ERIC und CENTRAL im November 2013 durchgeführt. Das Verzerrungspotential der eingeschlossenen Studien wurde anhand des Cochrane risk of bias Tools bewertet.

**Ergebnisse:** Fünf Studien erfüllten die Einschlusskriterien. Es wurden verschiedene in Gruppen durchgeführte Anti-Stigma-Interventionen sowie eine Broschüre untersucht. Die Intensität und Güte der Durchführung der meisten Interventionen war hoch. Vier Studien lieferten ausreichende Daten für eine statistische Analyse und zwei Studien wurden als ausreichend homogen bewertet, um sie für den Endpunkt „Selbststigma“ in einer Meta-Analyse zusammenzufassen. Die Meta-Analyse fand keinen signifikanten Effekt (standardisierte Mittelwertsdiffererenz [95%-Konfidenzintervall] nach 3 Monaten: –0.26 [–0.64, 0.12], I^2^=0%, n=108). Vier von Fünf Studien hatten ein hohes Verzerrungspotential. Die Qualität der Evidenz für die Interventionen und Endpunkte war sehr niedrig. Keine der Studien fand einen nachhaltigen Effekt für einen der untersuchten Endpunkte, einschließlich Genesung, Hilfesuchverhalten und Selbststigma.

**Schlussfolgerungen:** Die Wirksamkeit von Interventionen zur Reduktion von Selbststigmatisierung ist ungewiss. Bisherige Studien haben ein hohes Verzerrungspotential, verfügen nicht über eine ausreichende statistische Macht und verwenden Endpunkte, deren Eignung für die Evaluation solcher Maßnahmen teilweise fraglich ist. Zukünftige Studien sollten robuste Methoden verwenden und bei der Entwicklung der Maßnahmen auch praktischen Implikationen berücksichtigen wie die Relevanz, Implementierbarkeit und Verortung in der Routineversorgung.

## Introduction

Reducing the stigmatisation towards people with mental illnesses is one of the major goals of the World Health Organization’s Mental Health Action Plan [1]. Stigma occurs when “elements of labelling, stereotyping, separation, status loss, and discrimination occur together in a power situation that allows them” [2]. The negative consequences are far-reaching. Self-stigma can diminish people’s self-esteem and self-efficacy and negatively affect emotional well-being and personal relationships [3], [4]. It can have a negative impact on help-seeking behaviour, treatment adherence, and recovery [5], [6], [7]. The number of people who report some degree of self-stigma is significant. A recent systematic review of self-stigma in people with schizophrenia spectrum disorders found a self-stigma prevalence rate of 49%, highlighting the importance of the issue [8]. Another study estimated that self-stigma occurs in about 22% of people with bipolar disorder or depression [9].

Based on the aforementioned correlates of self-stigma, it is suggested that interventions reducing self-stigma may indirectly improve mental health related outcomes by improving help-seeking and adherence and empower people to attain their life goals. The following systematic review aims to investigate the benefits and harms of interventions for reducing or preventing self-stigma in people with a diagnosis of any mental illness treated in the community or as inpatients. 

### Background

Stigma can present on three levels [10]: Public or social stigma is defined as a negative reaction of the general population towards the stigmatised group based on stereotypes such as the belief that all people with mental illnesses are dangerous or incompetent. Structural or institutional stigma involves practices of private or public institutions that restrict the rights of stigmatised groups through rules, policies or processes. Self-stigma (also referred to as internalized stigma) occurs when people with mental illnesses accept the public stigma and incorporate it into their self-concept.

Interventions addressing self-stigma are usually complex in nature. They are, furthermore, embedded in the wider lives of people with mental illnesses and may interact with these – after all, stigma is (to some extent at least) socially constructed and resides in a larger context that includes social relationships, personal finances, treatments and other sources of support as well as patients’ educational, working and living environment, for example [11]. Based on fundamental cause theory, Hatzenbuehler and colleagues have argued that stigma may fulfil the criteria for a fundamental social cause, i.e. a social factor that remains associated with inequalities through the repeated production of mechanisms that link it with health [12]. 

Phelan and colleagues argue that stigma can have three functions: 1) domination (exploiting others and keeping them down in order to maintain wealth and power); 2) enforcement of social norms (making people comply with social norms); and 3) avoidance of disease (keeping people away) [13].

**Figure 1:**
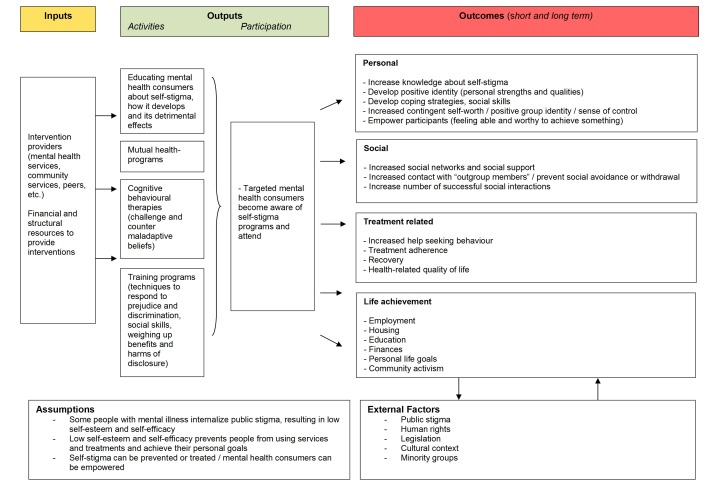
Logic model of interventions to reduce self-stigma in people with mental illnesses

Against this background, we developed a logic model in order to grasp how self-stigma is embedded in the social context of people with a mental illness and how it may be addressed (Figure 1 (Fig. 1)). This helped us to operationalize the research question of this review, specify the inclusion criteria and interpret the findings in context. For the purpose of this review a simple approach was used by mapping essential intervention components into inputs, processes and outcomes (included literature is provided in the Attachment 1). 

## Material and methods

### Inclusion criteria

We included randomised controlled trials (RCTs) of people with a clinically verified diagnosis of mental illness according to the DSM or ICD. We did not restrict studies by setting. Any intervention with the aim of reducing or preventing self-stigma was considered. Candidate interventions included psychoeducational interventions, cognitive behavioural therapy (CBT), mutual health programs (peer support interventions) as well as specific programs such as “coming out proud” and narrative enhancement and cognitive therapy (NECT) (see Attachment 1). All types of control interventions were allowed, including waiting list controls, attention controls, no intervention controls, and active controls.

Outcomes were drawn from the proposed logic model. They were divided into treatment-related outcomes (e.g. recovery, treatment adherence, quality of life); social outcomes (e.g. vocational or educational success, experienced stigma); personal (psychological) outcomes (e.g. self-stigma, perceived stigma, self-efficacy, self-esteem), life achievements (e.g. employment, education) and adverse effects. Length of follow-up was categorized into short-term (<3 months), medium-term (3–6 months) and long-term (>6 months). 

### Search strategy and selection of studies

Medline, Embase, PsycINFO, ERIC, and the Cochrane Controlled Trials Register were searched using a combination of search terms related to mental illnesses and self-stigma in combination with a validated filter for RCTs, where available [14]. In addition, reference lists of included studies and previous reviews were inspected for further potentially relevant articles. The WHO International Clinical Trials Registry Platform (ICTRP) and the NIH clinical trial database (https://clinicaltrials.gov/) were searched for ongoing studies. The search strategy was developed using a conceptual approach [15]. A sample search strategy for Medline is provided in the attachment. Searches were conducted in November 2013. In May 2014 we checked whether any studies identified through study registries had been published. We did not restrict searches to specific languages. 

Titles and abstracts of the initial search results were screened for potentially relevant studies by the primary reviewer (RB). A second reviewer (MM) screened a random 20% sample of these as a means of quality assurance. Full texts of potentially relevant studies were retrieved and assessed for eligibility independently by both reviewers. Disagreements were resolved through discussion. 

### Data extraction and assessment of included studies

Individual study data were extracted into standardized sheets using Covidence web software and included information on study ID, patient and intervention characteristics, outcome data and risk of bias items. Statistical data and information on risk of bias items were extracted by one reviewer (RB) and checked for accuracy by a second reviewer (MM). Disagreements were resolved through consensus. The extracted data were exported into Review Manager 5.2 for statistical analysis. Risk of bias was assessed in accordance with the Cochrane risk of bias tool [14]. Additional information was sought from the authors of the included studies where the published reports did not provide sufficient information. 

### Data analysis

It was planned to conduct meta-analyses for each group of interventions (e.g. interventions based on CBT methods versus waiting list control), if studies were deemed to be sufficiently homogeneous to be pooled statistically and in terms of clinical aspects. Data were considered statistically too heterogeneous for pooling if I^2^ was above 50% accompanied by a significant Chi^2^ test (p<0.1). We used random-effects models (REMs) in meta-analyses because we expected interventions which were at least somewhat heterogeneous. Continuous data derived from the same scale were analysed as differences in means (MD). If different scales were used to measure the same construct (e.g. self-stigma or quality of life), and the scales were deemed similar enough to be pooled, standardised mean differences (SMD) were calculated. For binary outcomes we calculated odds ratios. We attempted to conduct statistical analyses based on the intention to treat (ITT) principle, i.e. all participants were analysed in the group to which they were allocated. Where study reports only provided results from a per-protocol analysis, we contacted authors to obtain data from the ITT population. If studies failed to report information on variances for group means, these were calculated from p values, t values or confidence intervals in accordance with the methods described in the Cochrane Handbook [16].

## Results

**Figure 2:**
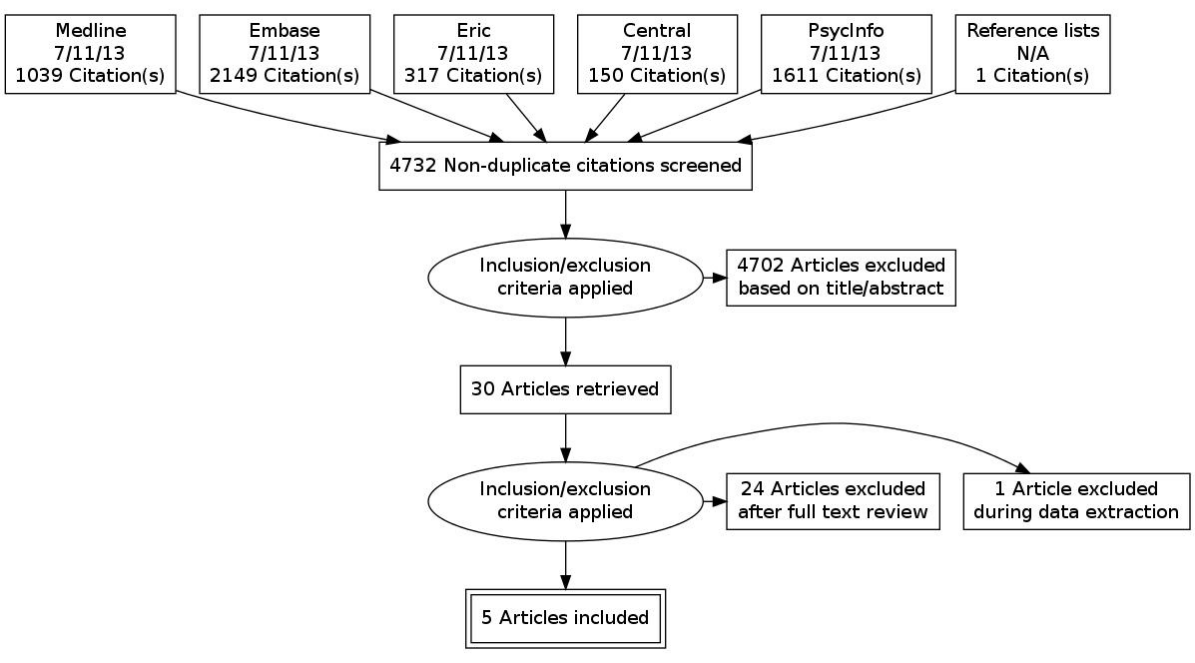
Flow chart of study selection process

The initial electronic database searches resulted in 5,266 potentially relevant references. After duplicates were removed electronically in EndNote, 4,731 references remained. Of these, 4,702 were excluded based on their titles and abstracts. Thirty studies were considered to potentially fulfil the eligibility criteria and retrieved for full text screening. Twenty-four of these studies were excluded after the predefined inclusion criteria were applied. One additional study was excluded, because it was not considered to be a genuine randomized trial during the data extraction process [17]. An additional potentially relevant study was identified by screening the reference lists of the included articles, but failed to fulfill the inclusion criteria. Seven on-going studies considered to be potentially eligible for this systematic review were identified from ICTRP and the clinicaltrials.gov. The publication status of these trials was checked before publication. Two of the registered trials had been published by this time, but neither of them fulfilled the inclusion criteria [18], [19]. The search process is documented with a flow chart in accordance with the PRISMA statement (Figure 2 (Fig. 2)) [20]. A list of excluded studies with reasons for exclusion is included in the Attachment 1. 

### Characteristics of included studies

Five randomized trials were included [21], [22], [23], [24], [25]. All included studies were two-arm randomized controlled trials with an inactive or attention control published within the last decade. 

The trials studied a variety of interventions. Four of the interventions were self-stigma reduction programs consisting of a series of group sessions. The specific interventions differed in their content and intensity. While the interventions were heterogeneous in nature, there was also some overlap: all of them included elements of psychoeducation and either CBT techniques or methods aimed at helping participants to develop a personally helpful identity. 

One study was quite different to the aforementioned ones in terms of the intervention and objective [23]. It evaluated a brochure that aimed at reducing barriers to seeking treatment. While the other studies directly addressed internalized stigma, this study was concerned with perceived stigma in that it addressed beliefs participants held about the potential reactions of others towards seeking mental health treatment and how they believed mental illness was perceived at the societal level. Four studies had a maximum follow-up of three months, while the longest follow-up was 6 months in one study [21]. A detailed table of study characteristics, including information on the reported outcomes, is included in the Attachment 1.

### Results of quality assessment

**Figure 3:**
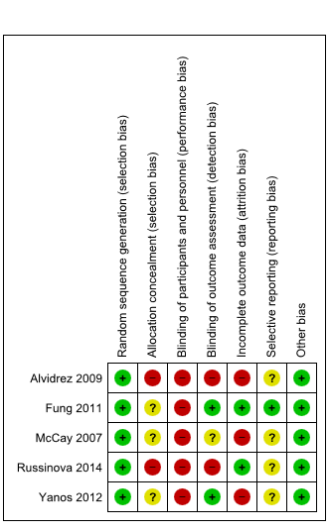
Summary of risk of bias assessments

Overall, the quality of reporting of the included trials was poor. Figure 3 (Fig. 3) summarizes the risk of bias assessments for each included study. Details including the rationale for the judgements per item and study are provided in the Attachment 1. Baseline characteristics of participants were not available separately for the intervention and control groups in two studies, and sample sizes per group and standard deviations were not always fully reported. Fortunately, the authors of three studies provided these data upon request.

### Effects of interventions

Due to the heterogeneity between the studies we present the results by interventions. Effect sizes for each study and outcome are summarized in Table 1 (Tab. 1). 

#### Meta-analytic effect of identity enhancing interventions on self-stigma

Two of the group-based intervention studies evaluated the effect of identity enhancing interventions, namely photovoice and narrative enhancement/cognitive therapy [24], [25]. These were the only two studies that we considered to be sufficiently homogeneous to be statistically pooled in that they both primarily included participants with a mix of DSM-IV diagnoses; evaluated a group intervention that included a combination of psychoeducation and techniques aimed at helping participants to develop personally meaningful identities; measured self-stigma on the Internalized Stigma of Mental Illness Scale (ISMI); and were statistically homogeneous (I^2^=0%, p>0.1). 

The ISMI is a self-report measure consisting of 29 items. Each item is measured on a 4-point Likert scale from 1 to 4, where a higher value denotes higher levels of self-stigma. The ISMI uses an index value that is calculated as the average value across all items. The ISMI was tested in an independent sample of mental health patients with various ICD-9 diagnoses including schizophrenia and depression and has good psychometric properties [26].

**Figure 4:**
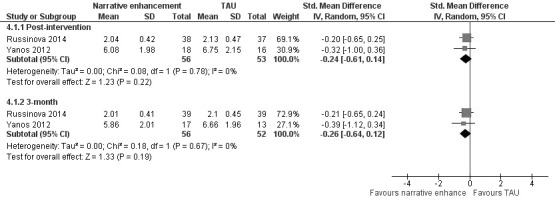
Meta-analysis of identity enhancing interventions

Russinova and colleagues used the ISMI as originally proposed, while Yanos and colleagues modified the instrument in a few aspects. Specifically, the stigma resistance subscale of the ISMI was reported separately. Possibly this was done because this subscale uses reverse-coded items, but the actual reasons were not reported. Furthermore, a Likert scale from 0 to 3 was used instead of the original Likert scale, which ranges from 1 to 4. The reasons for this were not reported. Lastly, the scores from the subscales were summed up in the NECT trial, while the photovoice study reported the average of the subscale scores, as the developers of the instrument have done in previous studies. Due to the differences in how the ISMI was used in the two studies, standardised mean differences (SMD) were calculated in the meta-analysis instead of using the original scale. The pooled results suggest a small, but statistically insignificant effect of the interventions on self-stigma (post-intervention: SMD –0.24 [–0.61, 0.14], 3-month: –0.26 [–0.64, 0.12]). A forest plot of the meta-analysis can be found in Figure 4 (Fig. 4).

#### Photovoice

Russinova and colleagues also assessed the effect of photovoice on empowerment and recovery. Empowerment was measured using the Empowerment Scale (ES). The ES consists of 28 items, each measured on a 4-point Likert scale from 1 to 4, where higher values indicate stronger feelings of empowerment. An index value is calculated by averaging the individual items. Thus, possible overall scores range from 1 to 4. The scale was developed for use with mental health consumers and appears to have good psychometric properties [27]. The instrument measures five domains: self-esteem/self-efficacy, power, community involvement and autonomy, optimism and righteous anger. There was no significant effect of photovoice on empowerment (post-intervention: MD 0.05 [–0.09, 0.19]; 3-month: MD 0.01 [–0.14, 0.16]). 

The effect of photovoice on recovery was measured on the Personal Growth and Recovery Scale (PGRS), a self-report outcome developed specifically for the study. The PGRS has 25 items, which are measured on a 4-point Likert scale, where a higher value indicates a higher level of perceived growth and recovery. The authors reported an index value, which was calculated as the average of the items (thus, the possible values for the outcome ranged from 1 to 4). While the authors report good psychometric properties, these results should be considered cautiously due to lack of evaluation in other cohorts. The study did not show a significant effect of photovoice on recovery immediately after the intervention or at three-month follow-up (post-intervention: MD 0.12 [–0.15, 0.39], 3-month: MD 0.21 [–0.03, 0.45]). 

#### Narrative enhancement/cognitive therapy (NECT)

Yanos and colleagues also measured the effect of NECT on self-esteem and quality of life. Self-esteem was measured on the Rosenberg Self-Esteem Scale (RSES). The instrument consists of 10 items measured on a 4-point Likert scale. The possible overall score ranges from 0 to 30, where a higher index value indicates higher levels of self-esteem. The RSES is a widely used measure of global self-esteem, which has been tested in a population of mental health patients and is considered to have good psychometric properties [28]. The study did not show an effect of NECT on self-esteem (post-intervention: MD 0.07 [–0.36, 0.50]; 3-month: MD –0.07 [–0.50, 0.36]). The sample had extremely low baseline values of self-esteem, however, suggesting a possible floor effect. 

Quality of life was measured on the Quality of Life Scale (QLS). The QLS is a therapist-reported instrument consisting of 21 items. Each item is measured on a 7-point Likert scale ranging from 0 to 6, where higher values indicate better functioning. The QLS was developed specifically for people with schizophrenia and measures four domains: relationships, occupational functioning, psychological functioning (sense of purpose, motivation etc.) and community participation [29]. An index value is calculated by adding up the average scores from the subscales. Thus, possible values range from 0 to 28. The QLS has good psychometric properties and is considered to be the gold standard for measuring health-related quality of life in people with schizophrenia [30]. There was no significant effect of NECT on quality of life (post-intervention: MD 2.80 [–6.78, 12.38]; 3-month: MD –4.74 [–16.18, 6.70]). The wide confidence interval includes both a potential beneficial or harmful effect. Note that the data on quality of life for this analysis come from the as-treated population, as the author did not provide data from the ITT population for this outcome.

#### Multi-faceted anti-stigma program

Fung and colleagues measured the effect of their program on self-stigma, self efficacy, treatment participation, and adherence. Self-stigma was measured using the Chinese version of the Self-Stigma in Mental Illness Scale (SSMIS). This scale consists of four subscales without an index value [31]. The subscales are ordered hierarchically in that they represent four progressive stages of stigma, namely stereotype agreement, stereotype awareness, self-concurrence and self-esteem decrement. Each subscale consists of 15 items rated on a scale from 1 to 9, where higher values indicate stronger self-stigma [32]. The possible index values for each subscale range from 15 to 135. Studies suggest acceptable psychometric properties for the SSMIS, although the internal consistency of the instrument is not well-established [31].

Due to the large number of subscales, the results for each SSMIS subscale are not presented here in full, but can be found in Table 1 (Tab. 1). In sum, there was no significant effect for any of the subscales at long-term follow-up. There was a relatively large baseline imbalance in favour of the control group for three of the subscales (9 to 14 points, data not shown). However, even when this is taken into account, the wide confidence intervals are compatible with no effect and preclude any meaningful conclusions. 

The effect of Fung et al.’s multi-faceted anti-stigma program on self-efficacy was measured with the Chinese General Self-efficacy Scale (CGSS). This instrument consists of 10 items measured on a 4-point Likert scale. The possible overall score ranges from 10 to 40, where a higher value indicates higher levels of self-esteem. The instrument has been tested with people with mental illness and has good psychometric properties [33]. Statistically significant effects were found post-intervention and at the 6-month follow-up, but not at intermediate time points (post-intervention: MD –4.02 [–7.08, –0.96]; 2-month: MD –0.17 [–2.83, 2.49]; 4-month: –2.43 [–5.39, 0.53]; 6-month: –3.12 [–6.04, –0.20]). 

The effect of the program on treatment participation and adherence were measured on two subscales of the Psychosocial Treatment Compliance Scale (PTCS). The PTCS is a therapist-reported outcome, which is used to rate attendance and adherence to various psychosocial treatments in the last three months. Attendance is based on 5 items measured on a 5-point Likert scale, where a higher value denotes better participation. Possible overall scores range from 5 to 25. Adherence is measured on twelve 5-point Likert scale items, where higher values denote better participation. Possible overall scores for adherence range from 12 to 60. A study in an independent cohort of people with a DSM-IV diagnosis of schizophrenia or other types of psychosis suggests good psychometric properties of the scale [34].

The study did not show a positive effect of the intervention on treatment attendance compared to an attention control group at any of the time points (post intervention: MD 1.12 [–0.49, 2.73]; 2-month: MD 0.50 [–1.01, 2.01]; 4-month: MD 0.56 [–0.83, 1.95]; 6-month MD 0.13 [–1.39, 1.65]). There was a significant effect of the anti-stigma program on treatment adherence post-intervention and at the 2-month follow-up, but these effects were not sustained (post-intervention: MD 3.52 [0.74, 6.30]; 2-month: MD 3.19 [0.17, 6.21]; 4-month: MD 2.79 [–0.20, 5.78]; 6-month: MD 1.90 [–1.55, 5.35]). 

#### Anti-stigma booklet

Alvidrez and colleagues measured the effect of their anti-stigma booklet on help-seeking behaviour, treatment adherence and perceived stigma. Help-seeking behaviour was measured as the rate of participants that entered treatment following provision of the booklet. No significant effect of the intervention on help-seeking behaviour was found (OR 1.46 [0.37, 5.80]). 

Treatment adherence was measured as the number of attended treatment sessions. There was no significant effect of the anti-stigma booklet on treatment adherence (3-month: MD 0.20 [–2.89, 3.29]).

Perceived stigma was measured using the Perceived Devaluation and Discrimination Scale (PDD), a self-complete measure consisting of 12 items. Each item is measured on a 6-point Likert scale, where higher values indicate higher levels of perceived stigma. The study provided a summary score by averaging the values from each item. The psychometric properties of the PDD are not well established [31]. There was no effect of the anti-stigma booklet on perceived stigma (3-month: MD 0.00 [–0.54, 0.54]). 

## Discussion

The systematic review found insufficient evidence on the effectiveness of interventions to reduce self-stigma in people with mental illness. It should not be concluded that interventions aimed at reducing self-stigma in people with mental illnesses are ineffective, however. The sparse evidence and a number of methodological reasons that reside in the limitation of the included studies might explain the disappointing results.

The heterogeneity of the studies, particularly in terms of interventions and outcomes, only allowed for one meta-analysis, resulting in low statistical power due to the small sample sizes of the included trials. Furthermore, the studies also have a number of methodological limitations. All but one trial were considered to be at high risk of bias, and none of the included studies was considered to have a low risk of bias. Performance bias was difficult to assess, since treatment-as-usual was rarely described in the studies and none of the studies included information on potential co-interventions.

The outcomes measures also provide a possible explanation for the lack of effect. Most of the scales that were used had acceptable psychometric properties in terms of reliability, internal consistency and content validity, for example. Their suitability for the evaluation of interventions is questionable, however, since most of them have been developed as survey measures, rather than for use in interventional research [31]. For example, information on possible floor and ceiling effects are missing for most of the scales, as well as evidence of whether they are responsive to change. The Rosenberg Self-Esteem Scale, for example, has been reported as being very unresponsive to change [35]. Furthermore, most of the instruments were based on 4-point Likert scales and thus may lack the ability to capture sufficient variability. 

A further limitation of the included studies is that none of them considered harms. Potential adverse effects of anti-stigma interventions include upsetting feelings, worsening of self-stigma, and negative effects of revealing the illness to others as a result of the intervention such as disapproval, exclusion or worry about not being treated as an equal. 

### Strengths and limitations

We used state of the art systematic review methods to identify, assess and synthesise evidence in order to avoid bias wherever possible. In order to make judgements as transparent as possible, we thoroughly documented and reported the review in accordance with PRISMA guidance. Some limitations of the reviews should be highlighted though. The primary reviewer (RB) screened all references, while only a 20% sample of the references was screened by a second reviewer (MM). Although the agreement between the reviewers was good (Cohen’s Kappa κ=0.71), it was not excellent. 

Decisions concerning intervention eligibility were not always clear cut. In particular, some interventions were developed in order to address specific aspects of mental illness related to self-stigma, such as self-esteem or empowerment (for example [36]). Since the aim of this review was to answer the question whether interventions specifically addressing self-stigma are effective, such studies were excluded on grounds that self-stigma was not the subject of the intervention. Lastly, publication bias is always a concern in systematic reviews – particularly in fields where studies are small and individual trials may have an important impact on the results. Due to the small number of studies and their heterogeneity it was not possible to examine publication bias statistically – for example using a funnel plot – or conduct corresponding sensitivity analyses. 

### Comparison with other reviews

Mittal and colleagues also acknowledged that the evidence for interventions against self-stigma is preliminary [37]. However, they suggested that some interventions had “promising” effects, in contrast to this review, which did not find any encouraging results. The authors did not use a stringent approach to analysing data and assessing risk of bias, which makes comparisons with this review difficult. The main reason for the discordant conclusion seems to lie in the underlying studies. Mittal and colleagues included studies of patients without a clear DSM or ICD diagnosis as well as a variety of study designs. In effect, the overlap in the evidence base between the reviews is small, which accounts for the somewhat discordant conclusions. 

Griffiths et al. pooled the results of four RCTs on self-stigma and did not find a significant effect (d=0.16; 95% CI: –0.41 to 0.73, p=0.57) [38]. Their meta-analysis was statistically highly heterogeneous, however (I^2^=74%). Yanos and colleagues conducted a narrative review of interventions addressing self-stigma [39]. As they did not provide any statistical data, it is difficult to make a comparison of the results of our reviews. However, they agree that much of the research on interventions addressing self-stigma is still in the early stages and that a deeper conversation about the aims, design and evaluation of interventions addressing self-stigma is required. A very recent meta-analysis by Tsang and colleagues found small, but significant effects in favour of interventions addressing self-stigma with standardised mean differences ranging from –0.33 to –0.43 in the pooled analyses [40]. The confidence intervals were wide, however, questioning their clinical importance. The quality of evidence is further weakened due to inclusion of unrandomised and uncontrolled studies. Furthermore, the authors pooled highly heterogeneous interventions ranging from intensive group programs addressing self-stigma to novel care models, making the meta-analyses difficult to interpret. While the authors do not consider the wider societal and care system implications of the interventions in contrast to our review, they agree that more and better quality research into interventions addressing self-stigma is warranted. 

### Avenues for further research

The studies included in this review were highly heterogeneous in both their intervention components and the composition of their participants. In order to successfully advance the intervention research on stigma reduction, we believe it would be helpful to take a step back and develop a sound theoretical base for the development of interventions and consider their active ingredients. This would ideally include the involvement of people who experience stigmatisation in order to better consider their needs. These might be sensitive to, for example, questions around the timing of such interventions (e.g. at the beginning of treatment or when the condition has stabilised; or when there is a risk of chronification or after discharge from an inpatient treatment) as well as the type of delivery (e.g. group-based or individually; through peers or professionals; in person or by other means). 

Furthermore, following Hatzenbuchler et al. and Phelan et al., reducing the negative effects of stigma is likely to require policies and interventions that address the social cause itself as well as the various mediators that link it to health, including self-stigma [11], [12]. The implication is that in order to address stigma, the motivation or power required to stigmatize others needs to be addressed.

A discussion is also needed about whether it makes sense to address self-stigma across many different mental health illnesses such as major depression, schizophrenia and social anxiety disorder. People with different mental health illnesses may have varying needs. These may also depend on other (potentially stigmatising) factors such as employment, gender and ethnicity. Lastly, we believe that it would be helpful to think about the differentiation between interventions specifically addressing self-stigma and other non-drug interventions, which may implicitly or explicitly address self-stigma, such as psychotherapy or self-help – and whether such a boundary is helpful. 

Future interventions aimed at addressing self-stigma should take into account the available literature in a systematic fashion, and involve key stakeholders in the interventions development process. Future studies should be based on adequate power calculations and be reported in accordance with current reporting guidelines for randomised trials and complex interventions. Outcomes should be defined in a participatory process together with people affected by mental illnesses to ensure that they reflect their needs. Public mental health professionals should not assume that interventions aimed at reducing self-stigma in people with mental illnesses will translate into better outcomes, if they are used without considering other contextual factors.

## Notes

### Acknowledgements

We did not receive funding for this work. We thank Jack Wilkinson for statistical advice and Philip Yanos and Jennifer Alvidrez for providing additional information on their studies. 

### Competing interests

This work was conducted as a master’s thesis at the University of Manchester. We declare that we do not have a conflict of interest. 

## Supplementary Material

Attachment 1

## Figures and Tables

**Table 1:**
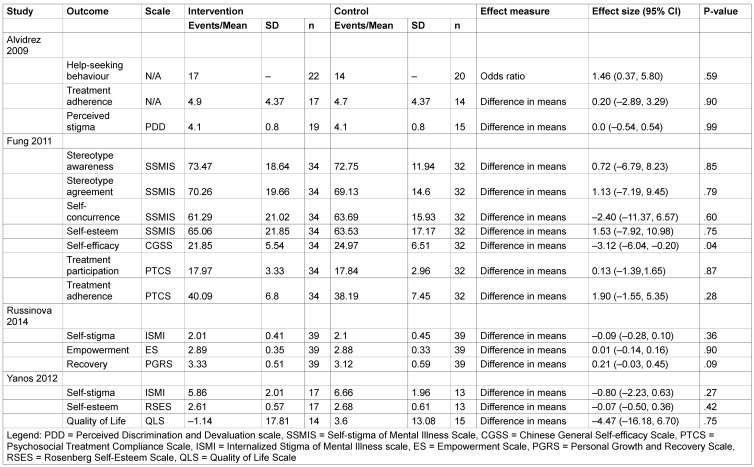
Effects of interventions at longest follow-up
