# Supplementary material for: Interventions for reducing self-stigma in people with mental illnesses: a systematic review of randomized controlled trials
Source: Ger Med Sci. 2017 Apr 24;15:Doc07. doi: 10.3205/000248 (PMC5404117; doi:10.3205/000248)
Supplement: Attachment 1 [file GMS-15-07-s-001.pdf]

Attachment

Literature for the logic model

Alvidrez J, Snowden LR, Kaiser DM. Involving consumers in the development of a psychoeducational booklet about stigma for Black mental health clients. Health Promot Pract 2010;11:249–58.

Anderson LM, Petticrew M, Rehfuess E, Armstrong R, Ueffing E, Baker P, et al. Using logic models to capture complexity in systematic reviews. Res Synth Methods 2011;2:33–42.

Brohan E, Slade M, Clement S, Thornicroft G. Experiences of mental illness stigma, prejudice and discrimination: a review of measures. BMC Health Serv Res 2010;10:80.

Corrigan PW, Larson JE, Rüsch N. Self-stigma and the "why try" effect: impact on life goals and evidence-based practices. World Psychiatry 2009;8:75–81.

Corrigan PW, Lundin RK. Coming out Proud to Eliminate the Stigma of Mental Illness. Manual for program facilitators and participants. Chicago, 2012.

Corrigan PW, Shapiro JR. Measuring the impact of programs that challenge the public stigma of mental illness. Clin Psychol Rev 2010;30:907–22.

Gerlinger G, Hauser M, De Hert M, Lacluyse K, Wampers M, Correll CU. Personal stigma in schizophrenia spectrum disorders: a systematic review of prevalence rates, correlates, impact and interventions. World Psychiatry 2013;12:155–64.

Link BG, Yang LH, Phelan JC, Collins PY. Measuring mental illness stigma. Schizophr Bull 2004;30:511–41.

Livingston JD, Boyd JE. Correlates and consequences of internalized stigma for people living with mental illness: a systematic review and meta-analysis. Soc Sci Med 2010;71:2150–61.

Lucksted A, Drapalski A, Calmes C, Forbes C, DeForge B, Boyd J. Ending self-stigma: Pilot evaluation of a new intervention to reduce internalized stigma among people with mental illnesses. Psychiatr Rehabil J 2011;35:51–4.

Yanos PT, Roe D, Lysaker PH. Narrative enhancement and cognitive therapy: a new group-based treatment for internalized stigma among persons with severe mental illness. Int J Group Psychother 2011;61:577–95.

Sample search strategy

A sample search strategy for Medline via Ovid:

1	mental health/
2	exp mental disorders/
3	mentally ill persons/
4	((mental* or psychiatr* or psychological* or developmental* or learning or substance*) adj (ill* or disorder* or disease* or distress* or disab* or problem* or health* or well-being or wellbeing or patient* or treatment or retardation)).tw.
5	((chronic* or severe* or serious* or persistent) adj (mental* or psychiatr* or psychological*)).tw.
6	(emotional adj3 (disorder* or problem*)).tw.
7	(((psychos#s or psychotic* or schizo* or depression or depressive or bipolar or mania or manic or obsessi* or panic or phobic or phobia or anorexi* or bulimi* or borderline or narcissis* or personality) adj1 disorder) or self injur* or self harm or substance abuse).tw.
8	1 or 2 or 3 or 4 or 5 or 6 or 7
9	stereotyping/
10	social distance/
11	self-stigma.mp.
12	(internali* or perceive* or personal or experienc*).mp.
13	stigma.mp.
14	12 and 13
15	9 or 10 or 11 or 14
16	8 and 15
21	(experiment* or intervention*).tw.
22	randomized controlled trial.pt.
23	controlled clinical trial.pt.
24	randomized.ab.
25	placebo.ab.
26	randomly.ab.
27	trial.ab.
28	groups.ab.
29	21 or 22 or 23 or 24 or 25 or 26 or 27 or 28
30	exp animals/ not humans.sh.
31	29 not 30
32	16 and 31

List of excluded studies

Adler AB, Bliese PD, McGurk D, Hoge CW, Castro CA. Battlemind debriefing and battlemind training as early interventions with soldiers returning from iraq: Randomization by platoon. J Consult Clin Psych. 2009;77(5):928-40.

Aho-Mustonen K, Tiihonen J, Repo-Tiihonen E, Ryynänen OP, Miettinen R, Räty H. Group psychoeducation for long-term offender patients with schizophrenia: an exploratory randomised controlled trial. Crim Behav Ment Health. 2011;21(3):163-76.

Anzai N, Yoneda S, Kumagai N, Nakamura Y, Ikebuchi E, Liberman RP. Training persons with schizophrenia in illness self-management: a randomized controlled trial in Japan. Psychiatr Serv. 2002;53(5):545-7.

Barnes E, Simpson S, Griffiths E, Hood K, Craddock N, Smith DJ. Developing an online psychoeducation package for bipolar disorder. J Ment Health. 2011;20(1):21-31.

Borras L, Boucherie M, Mohr S, Lecomte T, Perroud N, Huguelet P. Increasing self-esteem: efficacy of a group intervention for individuals with severe mental disorders. Eur Psychiat. 2009;24(5):307-16.

Corrigan PW, Sokol KA, Rusch N. The impact of self-stigma and mutual help programs on the quality of life of people with serious mental illnesses. Community Ment Health J. 2013;49(1):1-6.

Elafros MA, Mulenga J, Mbewe E, Haworth A, Chomba E, Atadzhanov M, et al. Peer support groups as an intervention to decrease epilepsy-associated stigma. Epilepsy Behav. 2013;27(1):188-92.

Farrer L, Christensen H, Griffiths KM, Mackinnon A. Web-based cognitive behavior therapy for depression with and without telephone tracking in a national helpline: secondary outcomes from a randomized controlled trial. JMIR. 2012;14(3):e68.

Griffiths KM, Christensen H, Jorm AF, Evans K, Groves C. Effect of web-based depression literacy and cognitive-behavioural therapy interventions on stigmatising attitudes to depression: randomised controlled trial. Br J Psychiatry. 2004;185:342-9.

Hammer JH, Vogel DL. Men's Help Seeking for Depression: The Efficacy of a Male-Sensitive Brochure about Counseling. Couns Psychol. 2010;38(2):296-313.

Hartman LI, Michel NM, Winter A, Young RE, Flett GL, Goldberg JO. Self-Stigma of Mental Illness in High School Youth. Can J Sch Psychol. 2013;28(1):28-42.

Henderson C, Brohan E, Clement S, Williams P, Lassman F, Schauman O, Dockery L, Farrelly S, Murray J, Murphy C, Slade M, Thornicroft G. Decision aid on disclosure of mental health status to an employer: feasibility and outcomes of a randomised controlled trial. Br J Psychiatry. 2013;203(5):350-7.

Klap R, Tang L, Schell T, Duan N, Wells K, Miranda J, et al. How quality improvement interventions for depression affect stigma concerns over time: a nine-year follow-up study. Psychiatr Serv. 2009;60(2):258-61.

Knight MTD, Wykes T, Hayward P. Group treatment of perceived stigma and self-esteem in schizophrenia: A waiting list trial of efficacy. Behav Cogn Psychoth. 2006;34(3):305-318.

Link BG, Struening EL, Neese-Todd S, Asmussen S, Phelan JC. On Describing and Seeking to Change the Experience of Stigma. Psychiatr Rehabil Skills. 2002;6(2):201-231.

Lucksted A, Drapalski A, Calmes C, Forbes C, DeForge B, Boyd J. Ending self-stigma: Pilot evaluation of a new intervention to reduce internalized stigma among people with mental illnesses. Psychiatric Rehabil J. 2011;35(1):51-54.

Michaels PJ, Corrigan PW, Buchholz B, Brown J, Arthur T, Netter C, et al. Changing stigma through a consumer-based stigma reduction program. Community Ment Health J. 2013;(Pagination):No Pagination Specified.

Morrison AP, Birchwood M, Pyle M, Flach C, Stewart SL, Byrne R, et al. Impact of cognitive therapy on internalised stigma in people with at-risk mental states. Br J Psychiatry. 2013;203(2):140-5.

O'Kearney R, Gibson M, Christensen H, Griffiths KM. Effects of a cognitive-behavioural internet program on depression, vulnerability to depression and stigma in adolescent males: a school-based controlled trial. Cognit Behav Ther. 2006;35(1):43-54.

Proudfoot J, Parker G, Manicavasagar V, Hadzi-Pavlovic D, Whitton A, Nicholas J, et al. Effects of adjunctive peer support on perceptions of illness control and understanding in an online psychoeducation program for bipolar disorder: A randomised controlled trial. J Affect Disorders. 2012;142(1-3):98-105.

Rüsch N, Abbruzzese E, Hagedorn E, Hartenhauer D, Kaufmann I, Curschellas J, Ventling S, Zuaboni G, Bridler R, Olschewski M, Kawohl W, Rössler W, Kleim B, Corrigan PW. Efficacy of Coming Out Proud to reduce stigma's impact among people with mental illness: pilot randomised controlled trial. Br J Psychiatry. 2014;204(6):391-7.

Shin SK, Lukens EP. Effects of psychoeducation for Korean Americans with chronic mental illness. Psychiatr Serv. 2002;53(9):1125-31.

Silverman MJ. Effects of music therapy on self- and experienced stigma in patients on an acute care psychiatric unit: a randomized three group effectiveness study. Arch Psychiatr Nurs. 2013;27(5):223-30.

Smith DJ, Griffiths E, Poole R, di Florio A, Barnes E, Kelly MJ, et al. Beating Bipolar: Exploratory trial of a novel internet-based psychoeducational treatment for bipolar disorder. Bipolar Disord. 2011;13(5-6):571-577.

Uchino T, Maeda M, Uchimura N. Psychoeducation may reduce self-stigma of people with schizophrenia and schizoaffective disorder. Kurume Med J. 2012;59(1-2):25-31.

Wade NG, Post BC, Cornish MA, Vogel DL, Tucker JR. Predictors of the Change in Self-Stigma Following a Single Session of Group Counseling. J Couns Psychol. 2011;58(2):170-182.

Wieczynski DM. Effects of a stigma management group for individuals with mental illnesses. PhD thesis. George Mason University; 2000.

Reasons for exclusion

**Study**	**Reason for exclusion**
Adler 2009	Did not include people with DSM or ICD diagnosis; intervention did not address stigma
Aho-Mustonen 2011	Intervention did not specifically address stigma
Anzai 2002	Intervention did not specifically address stigma
Barnes 2011	Not an RCT
Borras 2009	Intervention did not specifically address stigma
Corrigan 2013	Not an RCT
Elafros 2013	Not an RCT
Farrer 2012	Did not include people with DSM or ICD diagnosis
Griffiths 2004	Did not include people with DSM or ICD diagnosis
Hammer 2010	Did not include people with DSM or ICD diagnosis
Hartman 2013	Did not include people with DSM or ICD diagnosis
Henderson 2013	Did not include people with DSM or ICD diagnosis
Klap 2009	Intervention did not specifically address stigma
Knight 2006	Not an RCT

Study	Reason for exclusion
Link 2002	Did not include people with DSM or ICD diagnosis
Lucksted 2011	Not an RCT
Michaels 2013	Did not include people with DSM or ICD diagnosis
Morrison 2013	Did not include people with DSM or ICD diagnosis
O'Kearney 2006	Did not include people with DSM or ICD diagnosis
Proudfoot 2012	Intervention did not specifically address stigma
Rüsch 2014	Did not include people with DSM or ICD diagnosis
Shin 2002	Intervention did not specifically address stigma
Silverman 2013	Not an RCT
Smith 2011	Intervention did not specifically address stigma
Uchino 2012	Not an RCT
Wade 2011	Intervention did not specifically address stigma
Wieczynski 2000	Did not include people with DSM or ICD diagnosis

List of ongoing studies (potentially relevant)

Drapalaski AL. Reducing Internalized Stigma In People With Serious Mental Illness. Ongoing study. December 2010. Trial Registration: Clinicaltrials.gov NCT01259427

Gaebel W. Group Intervention for Improving Stigma Coping and Empowerment of People With Mental Illness (STEM). Ongoing study. June 2012. Trial Registration: Clinicaltrials.gov NCT01655368.

van Zelst C. Destigmatizing Mental Illness (D-STIGMI). Ongoing study. June 2011. Trial Registration: Nederlands Trial Register NTR2942.

Details of risk of bias assessment

Alvidrez (2009)

Bias	Authors judgement	Description according to study report, information provided by authors and rationale for judgement
Random sequence generation	Low risk	Not stated in publication. Additional information provided by author: "The randomization sequence was generated by an online random number generator."
Allocation concealment	High risk	*"After completion of the interview, the interviewer opened a sealed envelope to identify which type of information the client was randomly assigned to receive and delivered the appropriate information."*<br>According to additional information provided by the author the envelopes were opaque and placed in the recruitments and interview packets, which were sequentially numbered. However, the envelopes themselves were not sequentially numbered.
Blinding of participants and personnel	High risk	Blinding of participants and those delivering the treatment was not possible due to the nature of the intervention.
Blinding of outcomes assessment	High risk	*"Interviews and information sessions were conducted by two interviewers, one who was Black and one who was White. The interviewers alternated baseline and follow-up interviews for each client, so that clients would not be asked to provide their perceptions about the psychoeducation to the same interviewer who delivered it."*<br>Rationale for judgement: Outcomes were assessed by the same person delivering the intervention.
Incomplete outcome data	High risk	*"Thirty-four of the 42 clients (81%) completed follow-up interviews (19 Psychoeducation, 15 General Information). Of the eight not interviewed, 4 (50%) were contacted but could not be scheduled during the follow-up window, 3 (38%) were not successfully located, and 1 (13%) refused."*<br>Rationale for judgement: Data were missing from more than 10% of the participants. Data may not be missing completely at random, since it cannot be excluded that reasons for missing data are both unrelated to observable and unobservable variables. Thus, the available case analysis is likely to be biased. No imputation or sensitivity analysis were performed.
Selective reporting	Unclear risk	This was impossible to judge due to unavailability of a study protocol.
Other biases	Low risk	One of the scales was modified by the authors, which may influence validity. However, this outcomes was not relevant to the systematic review. No other potential biases were apparent.

Fung (2011)

**Bias**	**Authors judgement**	**Description according to study report, information provided by authors and rationale for judgement**
Random sequence generation	Low risk	*"The randomization of participants to the experimental or comparison protocol for each participating organization was conducted via the generation of random numbers ranging from 0.1 to 1.0 by SPSS. Individuals who received random numbers ≥0.5 were allocated to the experimental protocol and those who received random numbers <0.5 were allocated to the comparison group."*
Allocation concealment	Unclear risk	The method of concealment was not described in either of the two associated references and contacting authors was unsuccessful.
Blinding of participants and personnel	High risk	Blinding of participants and those delivering the treatment was not possible due to the nature of the intervention.
Blinding of outcomes assessment	Low risk	*"The CSSMIS, CAQ- SPMI, SUMD and CGSS were completed by experienced research assistants via face-to-face interview with the participants. The raters were not informed the treatment assignment of the participants."*
Incomplete outcome data	Low risk	*"The missing data was computed by the principle of Last Observation Carried Forward."*<br>*"The attrition rates for the experimental and comparison groups were 0% and 6.25% respectively."*<br>Rationale for judgement: LOCF may not have been appropriate because from the tables it appears that means were not stable across time and differential attrition rates suggests that data may not have been missing completely at random. However, data were missing from less than 15% of participants and differential attrition was less than 10%, rendering the risk of bias likely to be low (see section on incomplete outcome data for further explanations).
Selective reporting	Low risk	No study protocol was available, but the same outcomes were reported in the both study reports (PhD thesis and journal publication).
Other biases	Low risk	No other apparent biases were detected.

McCay (2007)

Bias	Authors judgement	Description according to study report, information provided by authors and rationale for judgement
Random sequence generation	Low risk	*"Participants were allocated to groups by randomly pulling group assignment."*<br>Rationale for judgement: Procedure is based on drawing lots principle and thus appears appropriate.
Allocation concealment	Unclear risk	*"Participants were allocated to groups by randomly pulling group assignment (group vs. control) from an envelope."*<br>Rationale for judgement: The characteristics of the envelopes were not reported, so the appropriateness of the method of allocation could not be judged. Contacting the author was unsuccessful.
Blinding of participants and personnel	High risk	Blinding of participants and those delivering the treatment was not possible due to the nature of the intervention.
Blinding of outcomes assessment	Unclear risk	No information on blinding of the outcome assessor was provided. Contacting the author was unsuccessful.
Incomplete outcome data	High risk	*"At baseline, 41 participants were randomly assigned to the treatment group and 26 participants to the control group. Two participants (one from each group) did not complete the data collection at 3 months (T2) and thus they were not included in this analysis; however, they remained in the study. Eighteen participants (26.9%, eleven in treatment group, seven in control group) dropped out of the study. The final sample described here includes 47 participants (treatment = 29, control = 18)."*<br>Rationale for judgement: Drop-out rates seem to be equal in both groups, but drop-outs appeared to have less severe disability than those remaining in the study, so data were probably not MCAR, rendering an available case analysis potentially biased. Sensitivity analysis was not conducted. The authors mention a lack of ITT analysis in the limitations section, but it is unclear whether this refers to handling of drop-outs or cross-overs between groups. Contacting the author was unsuccessful.
Selective reporting	Unclear risk	This was impossible to judge due to unavailability of a study protocol.
Other biases	Low risk	No other apparent biases were detected.

Russinova (2014)

**Bias**	**Authors judgement**	**Description according to study report, information provided by authors and rationale for judgement**
Random sequence generation	Low risk	*"A total of 82 individuals with serious mental illnesses enrolled at a university-based recovery center were randomly assigned to the antistigma photovoice program or to the wait-list, treatment-as-usual control group with the use of a computer-generated program that stratified on gender and racial-ethnic minority status."*
Allocation concealment	High risk	Allocation concealment was not reported in the study publication. Author contact clarified that participants were randomized based on the time they completed the self-reported baseline assessment and walked to the desk of the research staff to present their baseline packet. Thus, allocation was not concealed.
Blinding of participants and personnel	High risk	Blinding of participants and those delivering the treatment was not possible due to the nature of the intervention.
Blinding of outcomes assessment	High risk	Blinding of outcomes assessors was not reported in the study publication. Author contact clarified that the follow up assessments were administered by a research assistant working on the project who was aware of the group allocation of the participants.
Incomplete outcome data	Low risk	*"75 (92%) participants completed the posttest and 78 (95%) completed the three-month follow-up. Intent-to-treat analyses were conducted to test the antistigma photovoice program by using all available data regardless of extent of participation in antistigma photovoice."*<br>Rationale for judgement: Authors conducted an available case analysis. However, less than 10% of data were lost and differential attrition was less than 5% (for the 3 month follow up at least), rendering the risk of bias low (see methods section on incomplete outcome data for further information).
Selective reporting	Unclear risk	This was impossible to judge due to unavailability of a study protocol.
Other biases	Low risk	No other apparent biases were detected.

Yanos (2011)

Bias	Authors judgement	Description according to study report, information provided by authors and rationale for judgement
Random sequence generation	Low risk	*"Following the baseline interview, participants were randomized into either the experimental or control conditions using a computerized number generating system that assigned conditions based on client ID numbers."*
Allocation concealment	Unclear risk	Information on method of allocation concealment not reported in study publication. The method of allocation concealment remained unclear despite successful author contact.
Blinding of participants and personnel	High risk	Blinding of participants and those delivering the treatment was not possible due to the nature of the intervention.
Blinding of outcomes assessment	Low risk	The study author was contacted and confirmed that the interviewers were blind when the rating scales were being administered.
Incomplete outcome data	High risk	Only data from an "as treated" analysis based on exposure to the intervention were reported in the publication. The author provided additional data from the ITT population for two of the three outcomes included in this review.<br>Rational for judgement: data from more than 10% (up to 23%) of participants were missing at follow-up. Data were probably not missing at random. Sensitivity analyses were not conducted.
Selective reporting	Unclear risk	This was impossible to judge due to unavailability of a study protocol.
Other biases	Low risk	No other apparent biases were detected.

Characteristics of included studies

Author, year, reference, sample size	Study setting and inclusion criteria	Baseline characteristics	Intervention	Control	Outcomes	Fidelity of intervention delivery
Alvidrez 2009,[23]<br>n=42	**Setting:**<br>- Participants recruited from an outpatient clinical based in a county hospital<br>**Inclusion criteria:**<br>- English-speaking<br>- Black/African American<br>- First-time clients in outpatient mental health clinic<br>- Offered clinic services after intake<br>**Exclusion criteria:**<br>- Clients not offered services for other reasons (e.g.	**Diagnoses:**<br>Depression: 33 (78.6%)<br>PTSD: 16 (38.1%)<br>Other anxiety disorder: 15 (35.7%)<br>Pain disorder: 12 (28.6%)<br>Personality disorder: 4 (9.5%)<br>Co-occurring substance use disorder: 18 (42.9%)<br>**Demographics:**<br>Female: 29 (69.0%)<br>Age: mean 44.8 (SD 11.2)<br>Some high school education: 14 (33.3%)	**Psychoeducational information session based on booklet developed through extensive consumer involvement:**<br>- Sessions lasted between 15 and 30 minutes<br>- The booklet was developed based on qualitative interviews with Black mental health consumers regarding their experiences with mental health treatment and stigma (Title: "Getting Mental Health Treatment: Advice from People Who've Been There")<br>- The booklet included information on what consumers would have	**Psychoeducation al information session based on general information brochures:**<br>- Sessions lasted between 15 and 30 minutes<br>- Two standard brochures describing county mental health and outpatient services including information on referral and treatment were used	**Help-seeking behaviour**<br>- Number of participants entering treatment<br>**Treatment adherence:**<br>Number of treatment sessions attended<br>**Perceived stigma:**<br>Perceived stigma measured on the Perceived Devaluation and Discrimination Scale (PDD)	Not specifically reported, but since individual face to face sessions were used and the providers read out the booklet, it can be assumed that the interventions were delivered as planned.

Author, year, reference, sample size	Study setting and inclusion criteria	Baseline characteristics	Intervention	Control	Outcomes	Fidelity of intervention delivery
	already in treatment elsewhere)<br>**Setting:**<br>United States, outpatient mental health clinic based in a county hospital with services offered irrespective of insurance status	High school degree: 11 (26.2%)<br>Some college education: 15 (35.7%)<br>College degree: 2 (4.8%)<br>Disabled/not in job market: 30 (71.4%)<br>Working/looking for work/studying/retired: 12 (28.6%)<br><br>*Note: baseline characteristics were not reported separately for each arm of the trial*	liked to know before entering treatment, challenges and strategies they encountered regarding treatment adherence and advice they considered to be helpful for others (topics were chosen based on the most frequent themes in interviews)<br>- The booklet included quotes from interviews, was written in junior to high school reading level and could be read in 15 to 30 minutes<br>- Information on negative treatment experiences were omitted from the booklet			

Author, year, reference, sample size	Study setting and inclusion criteria	Baseline characteristics	Intervention	Control	Outcomes	Fidelity of intervention delivery
Fung 2011,[21] n=66	**Setting:**<br>- Community dwelling adults recruited from several psychiatric services in Hong Kong, China<br>**Inclusion criteria:**<br>- 18 to 65 years<br>- Diagnosed with DSM-IV schizophrenia<br>- Completed primary school education<br>- Received minimum of three months of psychosocial treatment before study<br>- Scoring ≥ 71.67 on stereotype	**Intervention group:**<br>Female: 16 (47.1%)<br>Age: 43.91 (SD 10.38)<br>Single: 23 (67.6%)<br>Married: 5 (14.7%)<br>Divorced: 6 (17.6%)<br>Living with family: 10 (29.4%)<br>Living alone: 9 (26.5%)<br>Living in hostel: 14 (41.2%)<br>Primary education: 8 (23.5%)<br>Secondary education: 22 (64.7%)<br>Tertiary education: 4 (11.8%)<br>Living on family income: 2 (5.9%)	**Self-stigma reduction program:**<br>- 16 session program consisting of 12 one-hour group and four 15-minute individual follow-up sessions<br>- The first two sessions included an introduction into the program and addressed concepts of recovery, information on prognosis and a session on confronting myths including a video of examples of people who have successfully recovered from schizophrenia<br>- Sessions 3 and 4 covered topics on personal experiences of social stigma, how feeling, thoughts and behaviours	**Newspaper reading group:**<br>- A newspaper reading group of the same intensity was used as an attention control<br>- No further information on this intervention were provided	**Emotional outcomes:**<br>Stereotype awareness (CSSMIS subscale)<br>Stereotype agreement (CSSMIS subscale)<br>Self-concurrence (CSSMIS subscale)<br>Self-esteem decrement (CSSMIS subscale)<br>Self-efficacy (CGSS)<br>**Treatment participation and adherence:**<br>These outcomes were measure through PTCS subscales	A detailed intervention manual was developed for the providers of the program. The principal investigator trained staff members using role play before the intervention was delivered. Interventions were delivered by qualified therapists with experience in working with people with schizophrenia and had completed a fidelity test (which was not further specified).

Author, year, reference, sample size	Study setting and inclusion criteria	Baseline characteristics	Intervention	Control	Outcomes	Fidelity of intervention delivery
	agreement, ≥ 64.94 on self-concurrence, or ≥ 64.06 on self-esteem decrement on the Chinese Self-stigma of Mental Illness Scale<br>**Excluded criteria:**<br>none reported	Disability allowance: 7 (20.6%)<br>Comprehensive Social Security Assistance: 25 (73.5%)<br>Others income: 0 (0.0%)<br>GAF score (0-100): 21.76 (SD 14.02)<br>BPRS score (18-126): 21.76 (14.02)<br>**Control group:**<br>Female: 13 (40.6%)<br>Age: 46.91 (SD 8.92)<br>Single: 26 (81.3%)<br>Married: 4 (12.5%)<br>Divorced: 1 (3.1%)<br>Living with family: 14 (43.8%)<br>Living alone: 6 (18.8%)	interact and how this can lead to self-stigma and impede recovery<br>- In sessions 5 to 7 participants learned how to combat self-stigma through techniques based on CBT and motivational interviewing<br>- Sessions 8 and 9 included a social skills training aimed at increasing assertiveness and dealing with stigma in social interactions; these sessions included different methods such as reflecting previous experiences and role play<br>- Sessions 10 and 11 were used to help participants identify realistic short- and long term personal goals and included the			

Author, year, reference, sample size	Study setting and inclusion criteria	Baseline characteristics	Intervention	Control	Outcomes	Fidelity of intervention delivery
		Living in hostel: 12 (37.5%)<br>Primary education: 13 (40.6%)<br>Secondary education: 17 (53.1%)<br>Tertiary education: 2 (6.3%)<br>Family income: 5 (15.6%)<br>Disability allowance: 6 (18.8%)<br>Comprehensive Social Security Assistance: 20 (62.5%)<br>Others income: 1 (3.1%)<br>GAF score (0-100): 26.88 (SD 12.47)<br>BPRS score (18-126): 26.88 (12.47)	development of a stepwise action plan and ways of evaluating success<br>- Session 12 was used as a round-up and consolidation session<br>- Individual follow-up sessions were used to discuss participants progress and coping strategies and provide further support and motivation			

Author, year, reference, sample size	Study setting and inclusion criteria	Baseline characteristics	Intervention	Control	Outcomes	Fidelity of intervention delivery
McCay 2007, [22]<br>n=65	**Setting:**<br>- Participants recruited from two first episode psychosis clinics in Toronto and Ottawa, Canada<br>**Inclusion criteria:**<br>- DSM-IV diagnosis of schizophrenia, schizophreniform disorder or schizoaffective disorder<br>- Aged 18 to 35<br>- Absence of previous psychiatric hospitalisations<br>- No antipsychotic medications received for more than eight weeks prior to the study	**Intervention:**<br>Female: 9 (31%)<br>Age: 25.1 (SD 4.86)<br>Length of education (years): 14.09 (SD 2.58)<br>Employment (hour per week): 6.52 (SD 5.17)<br>Living with parents: 19 (65.5%)<br>**Control:**<br>Female: 4 (22%)<br>Age: 26.2 (SD 7.03)<br>Length of education (years): 13.58 (SD 5.28)<br>Employment (hour per week): 5.28 (12.66)<br>Living with parents: 12 (66.7%)	**Group intervention:**<br>- 12 weekly group sessions, 90 minutes each<br>- Aimed at providing participants with healthy self-concepts<br>- Contents:<br>1) Express emotions related to the illness experience<br>2) Develop an acceptable perspective regarding personal illness experience<br>3) Develop sense of self beyond illness<br>4) Develop various coping strategies<br>5) Identify and facilitate pursuit of future goals	**Treatment as usual:**<br>Not further described	**Emotional outcomes:**<br>Self-Esteem (RSES)<br>Self-Efficacy (SES)<br>Perceived Stigma (LPSQ)<br>**Health-related outcomes:**<br>Quality of Life (QLS)<br>*Not: control group data were not reported in a way that allows extraction and statistical analyses*	A standardized group manual describing the content and process was developed to guide group sessions.<br>Two clinicians delivered each group intervention. Supervision and debriefing sessions were held regarding implementation of the manual.<br>No measures of fidelity were reported.

Author, year, reference, sample size	Study setting and inclusion criteria	Baseline characteristics	Intervention	Control	Outcomes	Fidelity of intervention delivery
	- Within two years of initial treatment (in hospital or out-patient setting) for a first episode of schizophrenia<br>- Ability to read, comprehend, and speak English<br>- Capacity to give informed consent to participate<br>**Exclusion criteria:**<br>- Drug-related psychosis<br>- Significant medical illness<br>- Organic brain syndrome					

Author, year, reference, sample size	Study setting and inclusion criteria	Baseline characteristics	Intervention	Control	Outcomes	Fidelity of intervention delivery
Russinova 2014, [24] n=82	**Setting:**<br>- Participants recruited from a psychosocial rehabilitation service based at a university<br>**Inclusion criteria:**<br>- Axis I or axis II DSM-IV diagnosis<br>- Marked functional impairment in social or occupational roles<br>- Age 18 or older<br>**Exclusion criteria:**<br>- not specified	Female: 56 (68%)<br>>40 years of age: 56 (68%)<br>White: 57 (70%)<br>African American: 7 (9%)<br>Asian: 3 (4%)<br>Hispanic: 9 (11%)<br>Unemployed: 69 (84%)<br>Bachelor’s degree or higher: 36 (44%)<br>Schizophrenia spectrum disorder: 28 (34%)<br>Bipolar disorder: 27 (33%)<br>Depressive disorder: 21 (26%)<br><br>*Note: Baseline characteristics were*	**Antistigma photovoice program:**<br>- Ten weekly 90 minute group sessions<br>- Peer-led<br>- Intervention combined photovoice technique, psychoeducation and exercises and was complemented with ongoing group discussions<br>- Aimed at reducing endorsement of stereotypes about mental illness<br>- In photovoice interventions participants take photos of everyday objects or events relevant to their lives. They then created narratives around the photos through group discussions facilitated	**Wait-list:**<br>- Treatment as usual, not further specified	**Emotional outcomes:**<br>Self-stigma (ISMI)<br>Empowerment (ES)<br>Recovery (PGRS)	Program was developed with involvement of peer leaders and consumers through an iterative process and then standardized. Content and process fidelity was measured based on 17 to 19 session specific items. On a 4-point rating scale, where 4 represents high fidelity, scores for content and process fidelity averaged 3.78 and 3.64, respectively.

Author, year, reference, sample size	Study setting and inclusion criteria	Baseline characteristics	Intervention	Control	Outcomes	Fidelity of intervention delivery
		not reported separately for intervention and control arm	through guided questions.<br>- All sessions involved elements of the photovoice techniques, including aspects of photojournalism, taking and discussion stigma related images, writing narratives and preparing a public display based on discussions on the appropriate target audience.<br>- Psychoeducation included the following topics: meaning and impact of stigma, prejudice and discrimination and coping strategies for dealing with stigma.<br>- Psychoeducation and photovoice were integrated and followed a			

Author, year, reference, sample size	Study setting and inclusion criteria	Baseline characteristics	Intervention	Control	Outcomes	Fidelity of intervention delivery
			three step approach: 1) weaknesses related to mental illness perceived by self or others were identified; 2) personal strengths were identified and 3) strengths and weaknesses were integrated in order to create a new, balanced self-perception.			
Yanos 2012, [25] n=39	**Setting:**<br>- Participants recruited from assertive community treatment programs in New York City and the VA center in Indianapolis, Indiana	**NECT:**<br>Female: 7 (33.3%)<br>Age: 47.14 (SD 7.86)<br>European-American: 3 (14.3%)<br>African-American: 17 (81%)<br>Hispanic: 1 (4.8%)<br>Education (in years): 11.04 (SD 2.13)	**NECT:**<br>- 20 one-hour structured group session based on four steps:<br>1) An introductory session that aims to elicit the degree of self-stigma through written exercises;<br>2) three weeks of psychoeducational including information on the prognosis of mental	**Treatment as usual:**<br>- All participants received standard care, including assessment, medication monitoring, case management, and rehabilitation services. Co-interventions addressing self-	**Emotional outcomes:**<br>Self-stigma (ISMI)<br>Self-esteem (RSES)<br>**Health-related outcomes:**<br>Quality of life (QLS)	Sessions were delivered by two out of a group of six therapists including clinical psychologists and clinicians who held Masters degree or were PhD candidates. A one day training was provided for them by three study investigators. The

Author, year, reference, sample size	Study setting and inclusion criteria	Baseline characteristics	Intervention	Control	Outcomes	Fidelity of intervention delivery
	**Inclusion criteria:**<br>- DSM–IV diagnosis of schizophrenia, schizoaffective disorder, bipolar disorder, or major depression<br>- scoring higher than a mean of 1.5 on the ISMI<br>- able to provide informed consent<br>**Exclusion criteria:**<br>- None reported	Age at first hospitalization: 20.9 (SD 6.49)<br>Schizophrenia: 6 (28.6%)<br>Schizoaffective: 10 (47.6%)<br>Bipolar I: 3 (14.3%)<br>Bipolar II: 1 (4.8%)<br>Major Depression: 1 (4.8%)<br>**Treatment as usual:**<br>Female: 44 (22.2%)<br>Age: 48.06 (SD 6.78)<br>European-American: 5 (27.8%)<br>African-American: 10 (55.6%)<br>Hispanic: 3 (16.7%)	illnesses, (self-)stigma and how it develops as well as common myths about mental illness;<br>3) eight sessions on cognitive restructuring including information on the interaction of thoughts, feelings and behaviour and various exercises on irrational beliefs and how to challenge them and<br>4) eight sessions with the task of constructing and sharing personally useful stories about oneself in general and in relation to one's illness.	stigma were not offered by either of the study sites.		training consisted of an overview of the intervention manual, and included role plays. Two supervisors provided one hour of supervision per group each week. They also assessed fidelity for one session from each intervention phase using a 5-point fidelity scale, where 5 was considered excellent. Fidelity across the two study sites ranged from 4 to 5 (mean 4.4).

Author, year, reference, sample size	Study setting and inclusion criteria	Baseline characteristics	Intervention	Control	Outcomes	Fidelity of intervention delivery
		Education (in years): 12.11 (SD 3.49)<br>Age at first hospitalization: 29.39 (SD 9.85)<br>Schizophrenia: 5 (27.8%)<br>Schizoaffective: 9 (50%)<br>Bipolar I: 2 (11.1%)<br>Bipolar II: 2 (11.1%)<br>Major Depression: 0				

Characteristics of measurement instruments used in the included studies

Study	Scale/variable	Self or therapist completed	Included domains	Number of items	Measurement scale	Method of index building	Range of possible values
Alvidrez 2009	Perceived Discrimination and Devaluation scale (PDD)	Self	(1) Perceived discrimination, (2) Perceived devaluation	12	6-point Likert scale	Mean of scores	1 to 6
Fung 2011	Self-stigma of Mental Illness Scale (SSMIS)	Self	(1) Stereotype awareness, (2) Stereotype agreement, (3) Self-concurrence, (4) Self-esteem	40	9-point Likert scale	Sum of scores	9 to 90
	Chinese General Self-efficacy Scale (CGSS)		(1) Personal self-efficacy, (2) Environmental self-efficacy	10	4-point Likert scale	Sum of scores	10 to 40
	Psychosocial Treatment Compliance Scale (PTCS)	Therapist	(1) Treatment participation, (2) Treatment adherence	17	5-point Likert scale	Sum of scores	17 to 85
Russinova 2014	Internalized Stigma of Mental Illness scale (ISMI)	Self	(1) Discrimination experience, (2) Alienation, (3) Stereotype endorsement, (4) Social withdrawal, (5) Stigma resistance	29	4-point Likert scale	Mean of scores	1 to 4
	Empowerment Scale (ES)	Self	(1) Self-esteem/Self-efficacy, (2) Power/Powerlessness, (3) Community activism and autonomy, (4) Optimism and control over future, (5) Righteous anger	24	4-point Likert scale	Mean of scores	1 to 4
	Personal Growth and Recovery Scale (PGRS)	Self	Information not provided (outcome measure developed for included study)	25	4-point Likert scale	Mean of scores	1 to 4

Study	Scale/variable	Self or therapist completed	Included domains	Number of items	Measurement scale	Method of index building	Range of possible values
Yanos 2012	Internalized Stigma of Mental Illness scale (ISMI)	Self	(1) Discrimination experience, (2) Alienation, (3) Stereotype endorsement, (4) Social withdrawal	29	4-point Likert scale	Mean of scores	0 to 3
	Rosenberg Self-Esteem Scale (RSES)	Self	Unidimensional measure	10	4-point Likert scale	Sum of scores	0 to 30
	Quality of Life Scale (QLS)	Therapist	(1) Interpersonal relationship,<br>(2) Instrumental role,<br>(3) Intrapsychic foundations,<br>(4) Common objects and activities	21	7-point Likert scale	Sum of scores	0 to 126
